# Does Far Transfer Exist? Negative Evidence From Chess, Music, and Working Memory Training

**DOI:** 10.1177/0963721417712760

**Published:** 2017-10-25

**Authors:** Giovanni Sala, Fernand Gobet

**Affiliations:** Department of Psychological Sciences, University of Liverpool

**Keywords:** chess, working memory, music, training, transfer

## Abstract

Chess masters and expert musicians appear to be, on average, more intelligent than the general population. Some researchers have thus claimed that playing chess or learning music enhances children’s cognitive abilities and academic attainment. We here present two meta-analyses assessing the effect of chess and music instruction on children’s cognitive and academic skills. A third meta-analysis evaluated the effects of working memory training—a cognitive skill correlated with music and chess expertise—on the same variables. The results show small to moderate effects. However, the effect sizes are inversely related to the quality of the experimental design (e.g., presence of active control groups). This pattern of results casts serious doubts on the effectiveness of chess, music, and working memory training. We discuss the theoretical and practical implications of these findings; extend the debate to other types of training such as spatial training, brain training, and video games; and conclude that far transfer of learning rarely occurs.

Transfer of learning is something all of us experience in our daily life. Knowledge of Samsung smartphones transfers to iPhones. Driving one’s car generalizes to other models of cars. Knowing how to cook spaghetti Bolognese is useful for cooking chicken pasta. All of these are examples of near transfer; that is, the generalization of a set of skills across two (or more) domains tightly related to each other. However, another type of transfer has attracted the attention of researchers for over a century: far transfer. Far transfer occurs when a set of skills generalizes across two (or more) domains that are only loosely related to each other (e.g., mathematics and Latin).

In a seminal article, Thorndike and Woodworth (1901) proposed their common-elements theory according to which transfer is a function of the extent to which two domains share common features. The theory predicts that while near transfer takes place often, far transfer is much less common. This point has been echoed by extensive research into the psychology of expertise and skill acquisition. For example, research on chess players has established that expert performance relies, to a large extent, on perceptual information such as the knowledge of tens of thousands of chunks (i.e., meaningful configurations of chess pieces; Chase & Simon, 1973; Sala & Gobet, 2017a). Because of its high specificity, such information is hardly transferable to other fields, as predicted by chunking theory (Chase & Simon, 1973) and template theory (i.e., an extension of chunking theory; Gobet, 2016; Gobet & Simon, 1996). However, research on expertise has also provided convincing evidence that experts—such as chess masters and professional musicians—possess, on average, superior overall cognitive ability. Importantly, domain-general cognitive abilities (e.g., intelligence, processing speed, and working memory [WM]) are reliable predictors of success for outcomes such as academic achievement (Deary, Strand, Smith, & Fernandes, 2007) and job proficiency (Hunter & Hunter, 1984).

At this point, we can see readers waving their hands: This evidence establishes correlation, but can we conclude that there is a causal relationship? Does training in cognitively demanding activities *make* people smarter? Is it possible to train domain-general cognitive abilities in one domain and hence obtain benefits in a vast number of areas? In other words, does far transfer occur?

This article presents the results of two meta-analyses investigating the cognitive correlates of expert performance and three meta-analyses on the effectiveness of cognitive training in the domains of chess, music, and WM training. Meta-analysis is a statistical method pooling together the results of all the studies available on a topic. Importantly, meta-analysis enables more reliable conclusions than a single experiment, because of the greater number of participants involved and the fact that studies are replicated. After the presentation of the five meta-analyses, we discuss the theoretical and practical implications of our findings and extend the discussion to other types of cognitive training.

## Does Far Transfer Occur? Insights From Chess, Music, and WM Training

### Comparison and correlational studies

People engaged in intellectual activities show superior overall cognitive ability compared with the general population. In the first of our meta-analytic reviews regarding the cognitive correlates of expert performance (Sala et al., 2017), we found that chess players’ overall cognitive ability was superior to that of non-chess players by half a standard deviation, a moderately large effect. When the focus shifted to the chess population and studies measuring the correlation between cognitive outcomes and chess skill, the pattern did not change. The second meta-analysis (Burgoyne et al., 2016) reported statistically significant correlations between chess skill and four broad measures of cognitive ability: (a) fluid intelligence—that is, the ability to solve new problems; (b) processing speed—that is, the efficiency of basic mental operations (e.g., as measured in reaction-time tasks); (c) short-term and WM memory—that is, the ability to retain, manipulate, and recall information over a brief period of time; and (d) comprehension knowledge—that is, the ability to use knowledge acquired through experience (e.g., vocabulary and reading comprehension). In other words, the more skilled the chess player, the higher his or her level of cognitive ability.

These results with chess players replicate previous findings on the role of cognitive ability in musicians. In Ruthsatz, Detterman, Griscom, and Cirullo (2008), a group of conservatory-level musicians got higher scores on Raven’s Progressive Matrices—a standard measure of fluid intelligence—compared with a group of novice musicians. Analogously, Lee, Lu, and Ko (2007) and Schellenberg (2006) found positive correlations between music skill, WM, and IQ.

### The hypothesis underlying the potential occurrence of far transfer

The positive correlation between cognitive ability and chess or music does not tell us anything certain about far transfer. An alternative explanation is that people with superior cognitive ability are more likely to engage and excel in chess and music. Moreover, given the research on expertise, one should be skeptical about the possibility of far transfer occurring.

So why do some researchers believe in the presence of far transfer from chess or music to other domains? The standard explanation assumes that these activities require domain-general cognitive abilities that may be trained by practice in a specific domain. Then, these enhanced cognitive abilities transfer to other domains. This idea has been made popular by an influential article by Jaeggi, Buschkuehl, Jonides, and Perrig (2008), which presented an experiment in which participants who received training on a WM task showed an improvement on a fluid intelligence test (Raven’s Matrices). A similar argument was deployed by Schellenberg (2006), according to whom music instruction enhances general intelligence, which in turn positively affects a wide range of other cognitive and academic abilities. In the same manner, Bart (2014) suggested that chess requires WM, fluid intelligence, and concentration capacity, and by practicing chess, children improve these abilities in general.

One theoretical foundation of the far-transfer hypothesis is neural plasticity—that is, the capability of the neural system of adapting and modifying under the pressure of the environment (Strobach & Karbach, 2016). Training cognitive function is thought to lead to changes in the neural system, which may account for the improvements on cognitive tests (Karbach & Schubert, 2013).

### Experimental studies

If these explanations are correct, training domain-general cognitive skills (WM, intelligence) through chess or music may transfer to other cognitive abilities and domain-specific skills (e.g., mathematics and literacy). We tested this hypothesis by running three meta-analyses, focusing on typically developing children and young adolescents.^1^ Children and young adolescents represent an ideal population on which to test the possible transfer of chess and music skills to other domains: During childhood and early adolescence, cognitive ability and academic skills are still at an initial stage of development, and thus, cognitive training is more likely to be effective than in adulthood.

The first two meta-analyses of the experimental studies (Sala & Gobet, 2016, 2017b) assessed the effect of chess and music instruction in enhancing children’s and young adolescents’ academic attainment (literacy and mathematics) and cognitive skills such as phonological processing, memory, and general intelligence. The third one (Sala & Gobet, 2017c) was carried out to evaluate the effects of WM training on academic achievement, fluid intelligence, and several measures of cognitive control (e.g., processing speed) in typically developing children. The results showed small to moderate overall effect sizes—that is, the overall quantitative measure of the effectiveness of the training—in all three meta-analyses (Table 1).^2^

**Table 1.** Results of the Three Meta-Analyses of the Experimental Studies

Training	Overall	Cognitive	Academic
*Chess*	0.34 [0.24, 0.44]	Overall: 0.33 [0.13, 0.53]	Mathematics: 0.38 [0.23, 0.53] Literacy: 0.25 [0.13, 0.37]
*Music*	0.16 [0.09, 0.22]	Intelligence (fluid/full-scale): 0.35 [0.21, 0.49] Memory: 0.34 [0.20, 0.48] Phonological processing: 0.17 [0.04, 0.29] Spatial cognition: 0.14 [–0.06, 0.34]	Mathematics: 0.17 [–0.02, 0.36] Literacy: –0.07 [–0.23, 0.09]
*Working memory*	0.12 [0.06, 0.18]	Fluid intelligence: 0.11 [–0.02, 0.24] Cognitive control: 0.09 [–0.08, 0.26]	Mathematics: 0.20 [0.03, 0.36] Literacy: 0.11 [0.00, 0.22]

*Note:* Results are presented as overall differences in standard deviations between treatment and control groups. The 95% confidence intervals are shown in brackets.

**Fig. 1.**
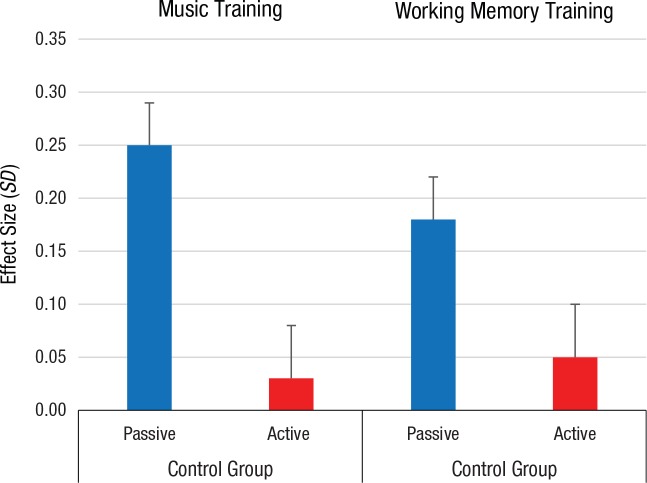
Results from the two meta-analyses of the experimental studies on music and working memory training: overall far-transfer effect sizes as a function of the type of control group (passive vs. active). Error bars represent standard errors.

Overall, these results may be considered “cautiously promising.” In fact, they are not. The size of the effects was inversely related to the quality of the experimental design. Specifically, when the experimental groups were compared with active control groups—other filler activities to rule out possible placebo effects such as positive expectations about the training (Boot, Simons, Stothart, & Stutts, 2013) and the excitement induced by a novel activity—the overall effect sizes were minimal or null. We focus on the WM- and music-treated samples, as the chess interventions included only one study with an active control group (with minimal effects: 0.10 *SD*). Regarding the effects of music instruction, the overall effect sizes were 0.25 *SD*, 95% CI = [0.17, 0.34], *k* = 64,^3^ and 0.03 *SD*, 95% CI = [−0.07, 0.12], *k* = 54, for the comparisons with passive and active control groups, respectively. About the effects of WM training, the overall effect sizes were 0.18 *SD*, 95% CI = [−0.09, 0.26], *k* = 34, and 0.05 *SD*, 95% CI = [−0.05, 0.15], *k* = 40, for the comparisons with passive and active control groups, respectively (Fig. 1).

The only exception to this pattern was the robust near-transfer effect that WM training exerted on other memory tasks (0.44 and 0.46 *SD* when the treated groups were compared with active and passive control groups, respectively).

### Research in other domains

Our results are in line with several recent studies on other types of cognitive training. In their systematic review, Simons et al. (2016) observe that no convincing evidence has been provided so far about the alleged generalized benefits of brain-training programs. Like WM training, such programs have been proven to enhance participants’ performance on the task they train and, at best, some other similar tasks. However, the studies with a strong experimental design (e.g., active control group and random allocation of the participants to the groups) showed no far-transfer effects.

Spatial ability has been found to be malleable by spatial training (Uttal et al., 2013). Considering that spatial ability predicts attainment in mathematics (Wai, Lubinski, & Benbow, 2009), one might expect that spatial training helps to develop mathematical ability. Regrettably, the attempts to obtain such a far-transfer effect have been unsuccessful so far (e.g., Xu & LeFevre, 2016).

Finally, Oei and Patterson (2015) have challenged the idea that action-video-game training can improve performance in a broad set of visuo-attentional and cognitive tasks. In their experiment, they used four different action video games as training tasks. These video games differed from each other in terms of their cognitive demands (e.g., different speed pace and level of selective attention). The results showed that participants’ improvements were limited to the cognitive abilities targeted by the game they played. This outcome is in line with Thorndike and Woodworth’s (1901) common-elements theory.

## Theoretical and Practical Implications

Together with recent experimental studies, our meta-analytic reviews have provided a clear pattern of findings: (a) People engaged in cognitively demanding activities such as chess and music have better overall cognitive ability than the general population; (b) cognitive ability (e.g., WM or general intelligence) is a predictor of chess skill and music skill, among many others; (c) training chess, music, or WM capacity does not reliably enhance any skill beyond the skills they train; and (d) far-transfer effects, when reported, probably stemmed from confounds such as placebo effects. The same pattern of results appears to occur with other types of cognitive training.

In accordance with Thorndike and Woodworth’s (1901) common-elements theory, far transfer remains a chimera. Consequently, theories assuming that skill acquisition and expert behavior rely on a large amount of domain-specific information—such as chunking (Chase & Simon, 1973) and template theories (Gobet & Simon, 1996)—find substantial corroboration in our results. In fact, these theories predict no (or minimal) far transfer from the training task to other nontrained tasks. Conversely, theories assuming that training domain-general cognitive abilities helps individuals to improve a broad range of domain-specific skills are not supported (for a review, see Strobach & Karbach, 2016).

Another theoretical implication of our results concerns neural plasticity. The substantial absence of far transfer suggests that the neural patterns observed in people engaged in cognitively demanding activities reflect modifications in domain-specific abilities (e.g., chess skill) rather than enhanced domain-general cognitive ability. The occurrence of specific neural patterns (anatomical and functional) and absence of far-transfer effects on cognitive tests have been reported in domains such as music (e.g., Tierney, Krizman, & Kraus, 2015), chess (e.g., Hänggi, Brütsch, Siegel, & Jäncke, 2014), and video game training (e.g., Colom et al., 2012).

In addition to theoretical aspects, the most obvious practical implications of our findings concern education. If skills rarely generalize across different domains, then the most effective way to acquire a skill is to train that particular skill. Considering the insights provided by the research on expert performance and cognitive training, educational and professional curricula should focus on discipline-related material rather than general principles without any specific reference to a particular subject (e.g., domain-general problem-solving skills). Moreover, the benefits of such domain-specific training should not be expected to generalize to other domains (e.g., learning Latin to improve logical thinking in mathematics).

Also, in line with the idea that training domain-general cognitive skills leads to benefits in a wide range of real-life skills, the last decade has seen the rise of a multibillion-dollar industry of commercial brain-training programs. Companies such as Posit Science and Cogmed claim that their training programs can help people in their daily, professional, and academic lives. However, in light of the results reported in the present paper, the effectiveness of these programs remains doubtful (see also the discussion in Simons et al., 2016).

## Conclusions and Future Directions

The meta-analytic reviews presented in this article strongly suggest that the optimism about the far-transfer effects of cognitive training is not justified, at least with typically developing children and young adolescents.^4^ Although cognitive ability correlates with domain-specific skills—for example, smarter people are more likely to be stronger chess players and better musicians—there is little evidence that chess or music instruction makes people smarter. Rather, smarter individuals are more likely to engage and excel in these fields. Moreover, converging evidence supporting Thorndike and Woodworth’s (1901) common-elements theory comes from the research on other types of training (e.g., WM training, video games, spatial training, and brain training) and expertise acquisition.

Future interventions trying to obtain far-transfer effects should strive for an experimental design including pretests and at least two control groups (a do-nothing group and an active control group). Such a design is the minimum standard in order to evaluate whether the putative benefits of cognitive training are genuine and not produced by statistical artifacts (e.g., differences at baseline level) and nonspecific factors (e.g., placebo effects, expectations). Another central aim is to identify the specific characteristics of the training that might improve one’s cognitive ability, which abilities they boost, and why these abilities should foster other nontrained abilities (i.e., far transfer).

Nonetheless, given the scarceness of evidence for far transfer in the literature, our prediction is that future experiments will show findings in line with those presented in this article. For this reason, researchers and policymakers should seriously consider stopping spending resources for this type of research. Rather than searching for a way to improve overall domain-general cognitive ability, the field should focus on clarifying the domain-specific cognitive correlates underpinning expert performance.

## Notes

1. Two studies in the meta-analysis about chess instruction included participants with learning disabilities and below-average IQ.

2. According to a common categorization (Cohen, 1988), effect sizes of 0.20, 0.50, and 0.80 are considered small, medium, and large, respectively.

3. *k* refers to the number of total comparisons between experimental and control groups in the meta-analytic model.

4. It is worth noting that this conclusion does not necessarily apply to the population of older adults. The aim of cognitive training in the elderly is mainly to slow down cognitive decline, not to enhance cognitive abilities. For more details, see Karbach and Verhaeghen (2014) and Melby-Lervåg and Hulme (2016).
